# Supplementary material for: Discriminatory Brain Processes of Native and Foreign Language in Children with and without Reading Difficulties
Source: Brain Sci. 2022 Dec 30;13(1):76. doi: 10.3390/brainsci13010076 (PMC9856413; doi:10.3390/brainsci13010076)
Supplement: Supplementary file 1 [file brainsci-13-00076-s001.zip › brainsci-2058474-supplementary.pdf]

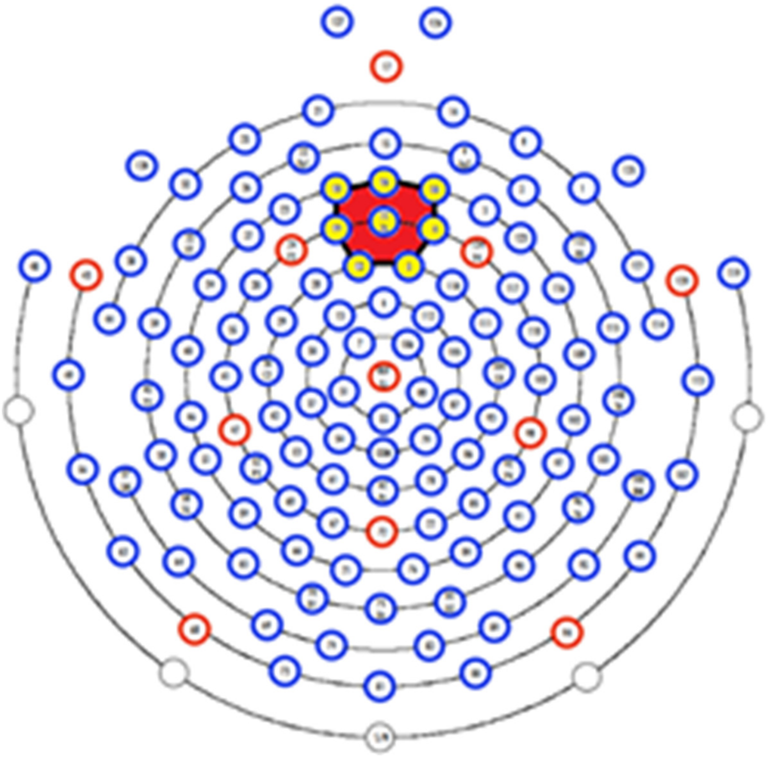

Figure S1: The electrodes selection (highlighted in yellow) and the region of interest for the ANOVA computations

Table S1: Number of accepted trials for each stimulus by group

				***CTR* group**				
Language		Finnish				English		
	Standards		Deviants		Standards		Deviants	
Stimuli	*suu pre-sii*	*suu pre-sai*	*sii*	*sai*	*shoe pre-she*	*shoe pre-shy*	*she*	*shy*
Range	[51–99]	[51–99]	[47–99]	[43–100]	[50–100]	[50–98]	[50–99]	[50–99]
Mean	82.52	83.12	83.01	85.65	81.24	81.47	82.23	82.04
SD	12.21	11.26	12.24	12.26	10.92	11.59	10.84	12.22
				***RD* group**				
Language		Finnish			English			
	Standards		Deviants		Standards		Deviants	
Stimuli	*suu pre-sii*	*suu pre-sai*	*sii*	*sai*	*shoe pre-she*	*shoe pre-shy*	*she*	*shy*
Range	[51–90]	[44–92]	[47–93]	[52–93]	[55–95]	[50–91]	[54–88]	[45–88]
Mean	76.30	76.15	75.84	76.73	75.19	74.07	74.03	73.96
SD	11.24	11.74	12.89	11.18	9.66	10.35	9.34	10.61

Table S2A. Summary of the stimuli used in the oddball paradigm presented in two blocks (Finnish and English) with the detailed trial numbers used in the experiment, the total duration of each stimulus (ms), and the frequencies (Hz) before and after the transition point from the consonant to the vowel

**Stimuli**	***Total duration of the stimuli (ms)***	***Trial number in the paradigm***	***Formant transition time point (from consonant to vowel) (ms)***	***Frequency at the formant transition voint (Hz)***	***Time (ms)***	***Max frequency before the transition point (Hz)***	***Time (ms)***	***Max frequency after the transition point (Hz)***	***Max Pitch***
**Finnish stimuli (Block 1)**									
suu	401	800	119	2,061	88	2,096	177	3,025	94.91
sai		100	119	1,930	89	1,739	183	2,811	94.90
sii		100	119	2,084	80	1,787	176	2,787	94.45
**English stimuli (Block 2)**									
shoe	401	800	120	2,096	77	1,596	172	2,608	95.58
shy		100	121	1,775	86	1,585	173	2,632	93.43
she		100	121	1,763	88	1,537	163	2,473	94.05

Note: The time point of change in frequency of the formant for the vowel immediately after the consonant is indicated in the *Formant transition time point* column.

Table S2B. Euclidean distance within language stimuli vowels

Finnish vowels following the /s/ sound

Vowel	*Type*	*F1 (Hz)*	*F2 (Hz)*	*F1 diff to [ʊ:]*	*F2 diff to [ʊ:]*	*Euclidean distance [ʊ:] to*
[ʊ:]	std	392	1205	-	-	-
[i:]	dev	311	2242	81	−1037	1040
[aɪ]	dev	573	1418	−181	−213	279

English vowels following the /ʃ/ sound

Vowel	*Type*	*F1 (Hz)*	*F2 (Hz)*	*F1 diff to [ʊ:]*	*F2 diff to [ʊ:]*	*Euclidean distance [ʊ:] to*
[ʊ:]	std	351	1715	-	-	-
[i:]	dev	309	2240	42	−525	526
[aɪ]	dev	586	1353	−235	362	795

Table S2C. Center of Gravity (COG) of the English [s] and Finnish [ʃ] fricatives within each stimulus

Fricative	*Stimuli*	*COG*	*Difference std – dev*
[s]	*suu*	6453	-
	sii	7557	−1104
	sai	6769	316
[ʃ]	*shoe*	5545	-
	she	5648	−103
	shy	4953	592

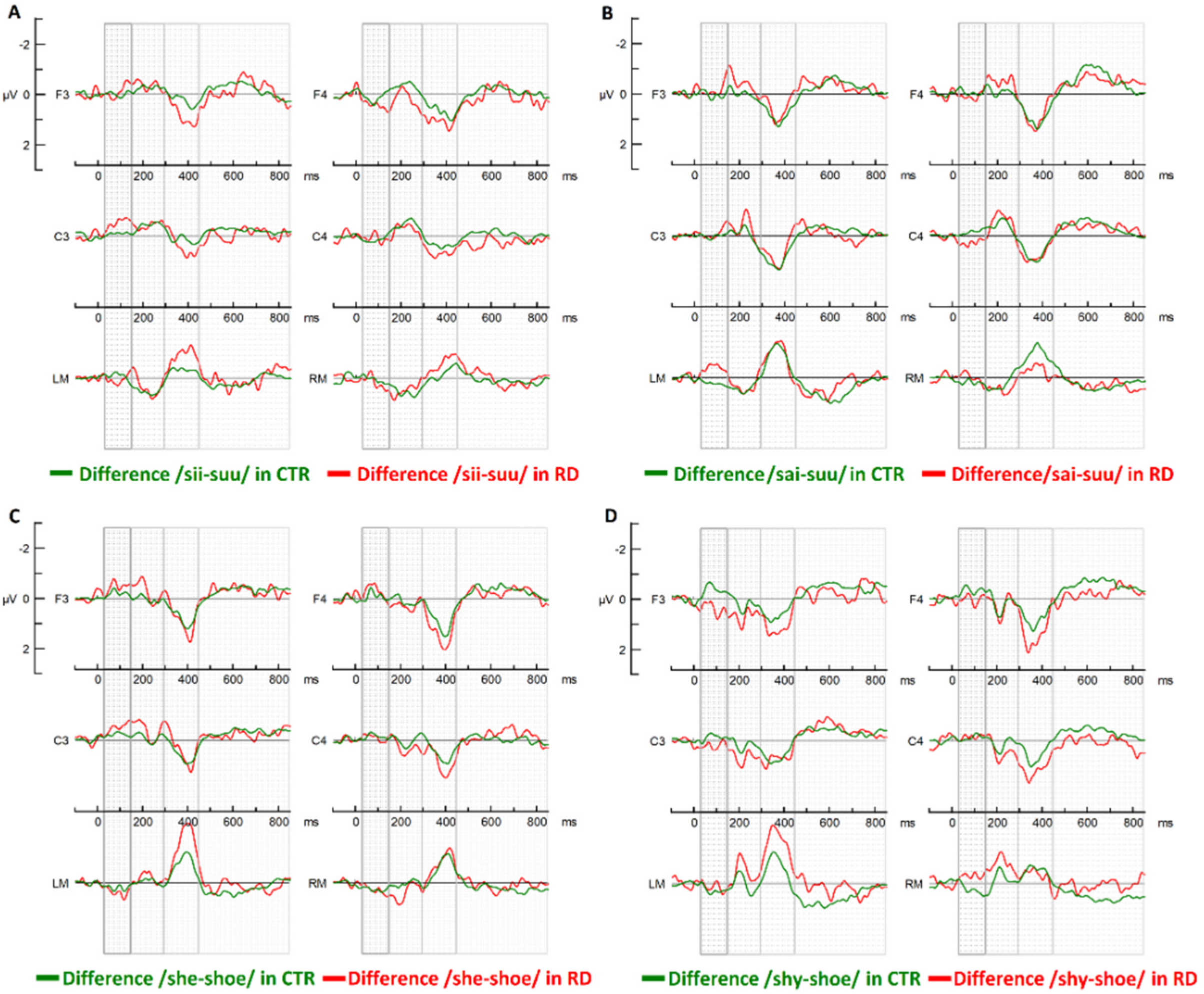

Figure S2. Group difference waveforms of the *RD* group (in red) vs the group difference waveforms of the *CTR* group (in green). (A) The difference waveforms in native *sii-suu* and (B) native *sai-suu*, (C) The difference waveforms in the foreign *she-shoe* and (D) in foreign *shy-shoe*.

Table S3. Summary of the ANOVA results

Condition

Condition	*Standard (suu/shoe)*	*Difference (sii/she)*	*Difference (sai/shy)*
MMN (150–300 ms)	F(1,110) = 8.349<br>p = 0.005*	F(1,110) = 2.824<br>p = 0.096	F(1,110) = 14.374<br>p = 0.000*
LDN (450–850 ms)	F(1,110) = 3.478<br>p = 0.650	F(1,110) = 8.972<br>p = 0.003*	F(1,110) = 2.322<br>p = 0.130

(Condition * Group)

Condition<br>*Time window*	*Standard (suu/shoe)*	*Difference (sii/she)*	*Difference (sai/shy)*
MMN (150–300 ms)	F(1,110) = 0.606<br>p = 0.438	F(1,110) = 3.145<br>p = 0.079	F(1,110) = 1.768<br>p = 0.186
LDN (450–850 ms)	F(1,110) = 0.310<br>p = 0.860	F(1,110) = 0.45<br>p = 0.833	F(1,110) = 0.798<br>p = 0.374
